# Association of single nucleotide polymorphisms in *CACNA 1A/CACNA 1C/CACNA 1H* calcium channel genes with diabetic peripheral neuropathy in Chinese population

**DOI:** 10.1042/BSR20171670

**Published:** 2018-05-28

**Authors:** Lin Sun, Jun Ma, Qian Mao, Yun-Long Yang, Lin-Lin Ma, Ling Niu, Li-Feng Liu

**Affiliations:** 1 Department of Endocrinology, The Affiliated Hospital of Beihua University, Jilin 132011, P.R. China; 2 Department of Radiology, The Affiliated Hospital of Beihua University, Jilin 132011, P.R. China; 3 Department of Thoracic Surgery, The Affiliated Hospital of Beihua University, Jilin 132011, P.R. China; 4 Department of Clinical Laboratory, The Affiliated Hospital of Beihua University, Jilin 132011, P.R. China; 5 Department of Stomatology, The Affiliated Hospital of Beihua University, Jilin 132011, P.R. China; 6 Department of Graduate, Beihua University, Jilin 132013, P.R. China

**Keywords:** CACNA 1A, CACNA 1C, CACNA 1H, Correlation, Diabetic peripheral neuropathy, Single nucleotide polymorphism

## Abstract

The present study was conducted to explore the correlations between single nucleotide polymorphisms (SNPs) in the calcium channel CACNA 1A, CACNA 1C, and CACNA 1H genes and diabetic peripheral neuropathy (DPN) amongst the Chinese population. In total, 281 patients diagnosed with type 2 diabetes participated in the present study. These patients were divided into the case group, which was subdivided into the DPN (143 cases) and the non-DPN groups (138 cases). Subsequently, 180 healthy individuals that had undergone routine health examinations were also recruited and assigned to the control group. PCR-restriction fragment length polymorphism (PCR-RFLP) was used to detect the genotype and allele frequencies of CACNA 1A, CACNA 1C, and CACNA 1H genes; logistic regression analysis to investigate the association of gene polymorphisms with DNP. Gene–gene interactions were then detected by generalized multifactor dimensionality reduction (GMDR). The results revealed that CACNA 1A rs2248069 and rsl6030, CACNA 1C rs216008 and rs2239050, and CACNA 1H rs3794619, and rs7191246 SNPs were all associated with DPN, while rs2248069, rsl6030, rs2239050, and rs7191246 polymorphisms were attributed to the susceptibility to DPN. It was also observed that the optimal models were three-, four- and five-dimensional models with a prediction accuracy of 61.05% and the greatest consistency of cross-validation was 10/10. In summary, these findings demonstrated that the SNPs in the CACNA 1A, CACNA 1C, and CACNA 1H genes were involved in the pathophysiology of DPN. In addition, polymorphisms in the CACNA 1A, CACNA 1C, and CACNA 1H genes and their interactions also had effects on DPN.

## Introduction

Diabetes mellitus (DM) refers to a group of metabolic disorders [1]. The primary complications that occur as a result of diabetes caused by blood vessel damage include damage to the nerves, eyes, and kidneys, which are clinically referred to as diabetic neuropathy, diabetic retinopathy, and diabetic nephropathy, respectively [2]. Diabetic neuropathy, which is also known as diabetic peripheral neuropathy (DPN), is the most common complication of diabetes that affects 25% of diabetic patients [3,4]. Glycemic control, cardiovascular risk management, and treatment for the reduction in pain and other symptoms are vital in the overall treatment of DPN patients [5]. Nonetheless, there is very little known in the clinical manifestations, the development and severity of DPN. This leads to a proposed hypothesis that genetic factors might play a role in the natural course of DPN [6]. Recent studies have also suggested that gene polymorphism might be an important risk factor in the development of DPN in patients with DM [7,8].

Calcium channels are specific ion channels that show selective permeability toward calcium ions, that consist of voltage-dependent calcium channels and ligand-gated calcium channels [9]. Located on chromosome 19p13, 17q22, and 12p13, respectively, CACNA 1A (P/Q-type), CACNA 1C (L-type), and CACNA 1H (T-type) are some of the most important calcium channel genes [10–12]. Mutations in the CACNA 1A and CACNA 1H genes highly affect the functions of calcium channels and might lead to absence seizures [13,14]. According to previous data, the rs216008 and the rs2239050 genotypes in CACNA 1C play a part in the development of DPN [15–17]. In addition, mutations of CACNA 1A and CACNA 1H are known to have abilities to process pain or disorder [18,19]. Genotypes of CACNA 1A rs2248069 and rsl6030 and CACNA 1H rs3794619 and rs7191246 were investigated recently for the efficacy of antiepileptic drugs, but were found without any associations [20]. So far, there are not a lot of studies that gave the single nucleotide polymorphisms (SNPs) in CACNA 1A, CACNA 1C, and CACNA 1H genes any emphasis in the development of DPN. Therefore, the present study was conducted with aims of investigating the relations of CACNA 1A rs2248069 and rsl6030, CACNA 1C rs216008 and rs2239050, and CACNA 1H rs3794619 and rs7191246 polymorphisms with DPN in order to provide sufficient evidence in determining the risk factors of DPN on a genetic basis.

## Materials and methods

### Study subjects selection process

In total, 281 patients with type 2 DM were recruited for the present study between the time periods of August 2014 and December 2017 from the Affiliated Hospital of Beihua University. Of the patients, 143 were grouped to the DPN group whereas 138 patients were assigned into the non-DPN group. The inclusion criteria were as follows: All DM diagnosis had been confirmed based on the World Health Organization (WTO) diagnostic criteria [21]: the patients presented with symptoms of DM; patients who had a random plasma glucose (PG) > 11.1 mmol/l (200 mg/dl) and fasting PG (FPG) > 7.0 mmol/l (126 mg/dl) or 2-h PG (2 h PG) > 11.1 mmol/l (200 mg/d1) during the oral glucose tolerance test (OGTT). Patients with type 1 DM were excluded from the present study.

In addition, a group of 180 healthy individuals who had undergone thorough physical examinations in the Affiliated Hospital of Beihua University during the same period were randomly selected as the control group. The exclusion criteria for patients in the control group included patients with: (i) history of smoking and alcohol or drug abuse; (ii) serious cardiovascular diseases or chronic disorders such as liver disease or renal insufficiency; (iii) acute complications of DM such as diabetic ketoacidosis; brain organic disease or nervous system disease; (iv) dysfunction in the central nervous system or other peripheral neuropathies caused by metabolic diseases; (v) recent trauma, surgery, or diagnosis of malignant tumor. There were no significant differences in age and gender between the case group and the control group (both *P*>0.05). The experiment was conducted in strict accordance with the Helsinki Declaration and has been approved by the Ethic Committee of the Affiliated Hospital of Beihua University. All participants had been given written informed consents prior to the experiment.

### DNA extraction and PCR-restriction fragment length polymorphism

A total of 2 ml venous blood was extracted from each patient following a 12-h fasting period. The blood samples were then transferred and stored in EDTA anticoagulation tubes and were sent to the labs to examine PG, insulin, C-peptide, liver and kidney function and blood lipid. The extraction of the DNA from the blood samples was carried out using a blood genome DNA extraction kit (Takara Biotechnology Co., Ltd, Dalian, China).

Prime 5.0 software was applied in the design of primers. The PCR-restriction fragment length polymorphism (PCR-RFLP) amplification primers of rs2248069 and rsl6030 in the CACNA 1A gene, rs216008, and rs2239050 in the CACNA 1C gene and rs3794619 and rs7191246 in the CACNA 1H gene were designed and synthesized by Shanghai Sangon Biological Engineering Technology Co., Ltd (Shanghai, China) (Table 1).

**Table 1:** Primer sequences of SNPs in CACNA 1A, CACNA 1C, and CACNA 1H genes for PCR-RFLP

Primer	Primer sequence	Annealing temperature	Annealing time	Cycle time
rs2248069	F: 5′-CCGAAAAGACTTCGACTCCG-3′	66°C	45 s	30
	R: 5′-TCCATCCCTGGGCCCCAGGA-3′			
rsl6030	F: 5′-ATAGGGAATGTCCACGACGC-3′	58°C	45 s	40
	R: 5′-GTGTGTTCTCACTTATAATG-3′			
rs216008	F: 5′-GACAAGTTTTAGCGGTACTT-3′	58°C	45 s	45
	R: 5′-AGGCCTACCTTGGGTTTGAA-3′			
rs2239050	F: 5′-TTGATAACTGGGGAGGAACT-3′	56.5°C	45 s	30
	R: 5′-GATGTCCGTTTTCTTCATTT-3′			
rs3794619	F: 5′-TTTAACGGTCTCACCGGGAT-3′	58°C	45 s	40
	R: 5′-ATTATCCAGTCTGGAAGAGT-3′			
rs7191246	F: 5′-TAGACTCGTTCCGAACAGTCT-3′	52°C	45 s	35
	R: 5′-ACACAAGCATACGGTCCCTC-3′			

Abbreviations: F, forward; R, reverse.

**Figure 1:**
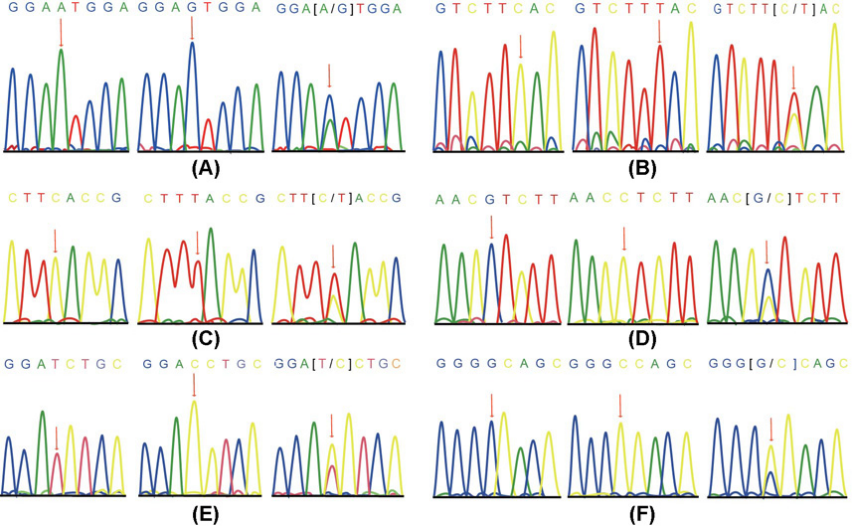
Allele sequence diagram of SNPs in *CACNA 1A/CACNA 1C/CACNA 1H* genes (**A**) CACNA 1A rs2248069; (**B**) CACNA 1A rsl6030; (**C**) CACNA 1C rs216008; (**D**), CACNA 1C rs2239050; (**E**) CACNA 1H rs3794619; (**F**) CACNA 1H rs7191246.

Then, 30 µl of the PCR-RFLP reaction system was used in the procedure and the mixture contained 0.2 μl DNA template, 3 μl 10× buffer (including magnesium), 1.2 ml dNTP mixture (2.5 mmol/l), 1.6 μl primer (0.8 μl each for forward and reverse primers), 0.5 μl TaKaRa Taq (5 U/μl), and 20.2 μl DEPC-treated water. Next, 1.5% agarose gel electrophoresis was used in the analysis of the PCR-RFLP amplified products, and the specific electrophoresis bands were sequenced (Figure 1).

### Statistical analysis

SPSS 19.0 integrated software (IBM Corp. Armonk, NY, USA) was applied for the analysis of the data and Hardy–Weinberg equilibrium was carried out to determine whether the samples were representatives of the group. Values with *P*≥0.05 indicated that the samples had reached genetic equilibrium and had a good group representation. The odds ratios (OR) and 95% confidence interval (CI) were calculated by univariate and multivariate logistic regression analysis in order to evaluate the correlation between SNPs of these genes with DPN. Categorical data were expressed as a percentage or rate, and were tested by χ^2^. Measurement data were presented as mean ± S.D. and *t* test was employed for comparison purposes. The generalized multifactor dimensionality reduction (GMDR) was used to analyze the interaction between multiple SNPs, to carry out sign test and permutation test, and to calculate the consistency of cross-validation and the accuracy of balance test for different factor combinations of each dimension. Multivariate logistic regression model was used for the verification of the gene–gene interaction in the optimal GMDR model, with *P*<0.05 considered as a statistically significant value.

## Results

### Baseline characteristics amongst the DPN, non-DPN, and control groups

The average course of disease in DPN patients was 12.41 ± 5.40 years. The course of disease, fasting insulin (FI), postprandial insulin (PI), and hemoglobin A1c (HbA1c) were significantly higher in the DPN group (all *P*<0.05) when compared with the non-DPN group. As for the levels of FPG, 2 h PG, HbA1c, FI, PI, and triglycerides (TG), there were non-significant differences between the DPN and control groups (all *P*<0.05). There were remarkably increased levels of FPG, 2 h FPG, HbA1c, FI, and PI in the non-DPN group in comparison with the control group (all *P*<0.05) (Table 2).

**Table 2:** Baseline characteristics of subjects amongst the DPN, non-DPN, and control groups

Baseline characteristics	DPN group (*n*=143)	Non-DPN group (*n*=138)	Control group (*n*=180)
Gender (M/F)	66/77	70/68	96/84
Age (years)	61.31 ± 10.16	60.19 ± 10.43	59.84 ± 9.05
Course of disease (years)	12.41 ± 5.40*^†^	6.34 ± 3.70*	0
BMI (kg/m^2^)	25.36 ± 3.29	25.38 ± 3.51	26.36 ± 5.14
FPG (mmol/l)	8.10 ± 2.19*	7.97 ± 2.48*	4.83 ± 0.54
2 h PG (mmol/l)	14.59 ± 4.56*	14.66 ± 4.35*	15.78 ± 3.17
FI	2.90 ± 0.91*^†^	2.03 ± 0.87*	1.21 ± 0.71
PI	4.00 ± 1.01*^†^	3.18 ± 1.03*	1.80 ± 0.46
Fasting C-peptide	1.25 ± 0.68	1.14 ± 0.57	1.13 ± 0.67
Postprandial C-peptide	1.92 ± 0.86	2.03 ± 0.86	1.99 ± 0.78
HbA1c (%)	8.47 ± 2.93*^†^	9.90 ± 2.98*	4.73 ± 1.17
TC (mmol/l)	4.95 ± 1.18	4.92 ± 1.77	4.93 ± 0.87
TG (mmol/l)	3.37 ±1.56*	3.68 ± 1.38*	2.06 ± 0.59
HDL-C (mmol/l)	1.06 ± 0.37*	1.14 ± 0.42*	1.41 ± 0.32
LDL-C (mmol/l)	3.11 ± 0.97*	2.93 ± 0.87	2.87 ± 0.74
APO-A (g/l)	1.33 ± 0.30	1.33 ± 0.41	1.26 ± 0.24
APO-B (g/l)	0.90 ± 0.30	0.87 ± 0.30	0.83 ± 0.28
Urea (mmol/l)	5.73 ± 1.99*	5.59 ± 1.86*	4.38 ± 1.27
Creatinine (mmol/l)	70.99 ± 20.64	76.70 ± 26.13*	69.48 ± 26.45
Uric acid (mmol/l)	320.03 ± 88.07*	317.38 ± 95.38*	376.66 ± 109.40

Abbreviations: APO-A, apolipoprotein A; APO-B, apolipoprotein B; BMI, body mass index; HDL-C, high-density lipoprotein cholesterol; LDL-C, low-density lipoprotein cholesterol; TC, total cholesterol. *, *P*<0.05 compared with the DPN group. ^†^, *P*<0.05 compared with the non-DPN group.

### Genotype and allele frequency distributions in the CACNA 1A, CACNA 1C, and CACNA 1H genes

The genotype and allele frequency distributions in the CACNA 1A, CACNA 1C, and CACNA 1H genes are illustrated in Table 3. Hardy–Weinberg equilibrium demonstrated that the distributions of genotype and allele frequencies of these genes reached genetic equilibrium (*P*>0.05), indicating that the population was well represented by the samples in the present study.

**Table 3:** Distributions of CACNA 1A, CACNA 1C, and CACNA 1H genotypes and alleles in the DPN, non-DPN, and control groups

Genotype	DPN group (%)	Non-DPN group (%)	Control group (%)	P*	OR (95% CI)	*P*^†^	OR (95% CI)	*P*^‡^	OR (95% CI)
**CACNA 1A**									
rs2248069									
AA	32 (22.4)	50 (36.2)	80 (44.4)		1		1		1
GA	68 (47.5)	72 (52.2)	87 (48.3)	0.012	1.95 (1.16, 3.28)	0.168	1.48 (0.85, 2.57)	0.243	1.32 (0.83, 2.12)
GG	43 (30.1)	16 (11.6)	13 (7.3)	<0.001	8.27 (3.93, 17.40)	<0.001	4.20 (2.03, 8.68)	0.1	1.97 (0.87, 4.44)
GA + AA	111 (77.6)	88 (63.8)	100 (55.6)	<0.001	2.78 (1.70, 4.54)	0.012	1.97 (1.17, 3.33)	0.14	1.41 (0.89, 2.22)
A	132 (46.2)	172 (62.3)	247 (68.6)		1		1		1
G	154 (53.8)	104 (37.7)	113 (31.4)	<0.001	2.55 (1.85, 3.52)	<0.001	1.93 (1.38, 2.70)	0.097	1.32 (0.95, 1.84)
rsl6030									
CC	80 (55.9)	85 (61.6)	125 (66.7)		1		1		1
CT	27 (18.9)	35 (25.4)	46 (28.3)	0.759	0.92 (0.53, 1.59)	0.507	0.82 (0.46, 1.48)	0.671	1.12 (0.67, 1.88)
TT	36 (25.2)	18 (13.0)	9 (5.0)	<0.001	6.25 (2.86, 13.67)	0.026	2.07 (1.08, 3.94)	0.01	2.94 (1.26, 6.86)
CT + TT	63 (44.1)	53 (38.4)	60 (33.3)	0.012	1.79 (1.13, 2.83)	0.336	1.26 (0.78, 2.03)	0.143	1.42 (0.89, 2.26)
C	187 (65.4)	205 (74.3)	291 (80.8)		1		1		1
T	99 (34.6)	71 (25.7)	69 (19.2)	<0.001	2.45 (1.70, 3.52)	0.022	1.53(1.06, 2.20)	0.015	1.60(1.09, 2.35)
**CACNA 1C**									
rs216008									
CC	54 (37.8)	69 (50.0)	110 (61.1)		1		1		1
CT	51 (35.6)	38 (27.5)	40 (22.2)	<0.001	2.60 (1.53, 4.40)	0.054	1.72 (0.99, 2.98)	0.128	1.51(0.89,2.59)
TT	38 (26.6)	31 (22.5)	30 (16.7)	0.001	2.58 (1.45, 4.60)	0.137	1.57 (0.86, 2.84)	0.093	1.65(0.92,2.96)
CT + TT	89 (62.2)	69 (50.0)	70 (38.9)	<0.001	2.59 (1.65, 4.07)	0.039	1.65 (1.03, 2.65)	0.048	1.57(1.00,2.46)
C	159 (55.6)	176 (63.8)	260 (72.2)		1		1		1
T	127 (44.4)	100 (36.2)	100 (27.8)	<0.001	2.08 (1.50, 2.88)	0.048	1.41(1.00, 1.97)	0.023	1.48(1.06,2.07)
rs2239050									
GG	67 (46.9)	82 (59.4)	115 (63.9)		1		1		1
GC	48 (33.5)	46 (33.3)	57 (31.7)	0.138	1.45 (0.89, 2.36)	0.354	1.28 (0.76, 2.14)	0.614	1.13 (0.70, 1.83)
CC	28 (19.6)	10 (7.3)	8 (4.4)	<0.001	6.01 (2.59, 13.94)	0.002	3.43 (1.55, 7.56)	0.253	1.75 (0.66, 4.63)
GC + CC	76 (53.1)	56 (40.6)	65 (36.1)	0.002	2.01 (1.28, 3.14)	0.035	1.66 (1.04, 2.67)	0.416	1.21 (0.77, 1.91)
G	182 (63.6)	210 (76.1)	287 (79.7)		1		1		1
C	104 (36.4)	66 (23.9)	73 (20.3)	<0.001	2.25 (1.58, 3.20)	0.001	1.82 (1.26, 2.62)	0.272	1.24 (0.85, 1.80)
**CACNA 1H**									
rs3794619									
TT	64 (44.8)	93 (67.4)	120 (66.7)		1		1		1
TC	55 (38.4)	32 (23.2)	41 (22.8)	<0.001	2.52 (1.52, 4.17)	<0.001	3.48 (1.94, 6.22)	0.979	1.01 (0.59, 1.72)
CC	24 (16.8)	13 (9.4)	19 (10.6)	0.011	2.37 (1.21, 4.65)	0.001	2.68 (1.27, 5.66)	0.747	0.88 (0.41, 1.88)
TC + CC	79 (55.2)	45 (32.6)	60 (33.3)	<0.001	2.47 (1.57, 3.88)	<0.001	2.55 (1.57, 4.14)	0.892	0.97 (0.60, 1.55)
T	183 (64.0)	218 (79.0)	281 (78.1)		1		1		1
C	103 (36.0)	58 (21.0)	79 (21.9)	<0.001	2.00 (1.42, 2.83)	<0.001	2.12 (1.45, 3.09)	0.777	0.95 (0.65, 1.39)
rs7191246									
GG	83 (58.0)	106 (76.8)	143 (79.4)		1		1		1
GC	30 (21.0)	21 (15.2)	30 (16.7)	0.062	1.72 (0.97, 3.06)	0.058	1.82 (0.97, 3.42)	0.854	0.94 (0.51, 1.74)
CC	30 (21.0)	11 (8.0)	7 (3.9)	<0.001	7.38 (3.11, 17.56)	0.001	3.48 (1.65, 7.36)	0.126	2.12 (0.80, 5.65)
GC + CC	60 (42.0)	32 (23.2)	37 (20.6)	<0.001	2.79 (1.71, 4.57)	0.001	2.40 (1.43, 4.01)	0.572	1.17 (0.68, 1.99)
G	196 (68.5)	233 (84.4)	316 (87.8)		1		1		1
C	90 (31.5)	43 (15.6)	44 (12.2)	<0.001	3.30 (2.21, 4.93)	<0.001	2.49 (1.65, 3.75)	0.222	1.33 (0.84, 2.09)

*, compared with the DPN group and the control group *P*<0.05. ^†^, compared with the non-DPN group *P*<0.05. ^‡^, compared with the non-DPN group and the control group *P*<0.05.

The results showed that there was a notable difference in the genotype and allele frequency distributions in the CACNA 1A, CACNA 1C, and CACNA 1H genes (all *P*<0.05). G allele of CACNA 1A rs2248069 (OR = 2.55, 95% CI: 1.85–3.52, *P*<0.05), T allele of CACNA 1C rs216008 (OR = 2.08, 95% CI: 1.50–2.88, *P*<0.05) and C allele of CACNA 1C rs2239050 (OR = 2.25, 95% CI: 1.58–3.20, *P*<0.05) were found to increase patients’ susceptibility to DPN. C allele of CACNA 1H rs3794619 (OR = 2.00, 95% CI: 1.42–2.83, *P*<0.05) and C allele of CACNA 1H rs7191246 (OR = 3.30, 95% CI: 2.21–4.93, *P*<0.05) were also contributing factors in the development of DPN (Table 3). Similar results were observed in the genotype and allele frequency distributions of CACNA 1A, CACNA 1C, and CACNA 1H genes between the DPN and non-DPN groups. G allele of CACNA 1A rs2248069, T allele of CACNA 1A rsl6030, T allele of CACNA 1C rs216008, C allele of CACNA 1C rs2239050, C allele of CACNA 1H rs3794619, and C allele of CACNA 1H rs7191246 were all attributed to an increased susceptibility to DPN (Table 3). For the genotype and allele frequency distributions of CACNA 1A, CACNA 1C, and CACNA 1H genes between the non-DPN and control groups, it was revealed that the T allele of CACNA 1A rsl6030 (OR = 1.60, 95% CI: 1.09–2.35, *P*<0.05) and T allele of CACNA 1C rs216008 (OR = 1.48, 95% CI: 1.06–2.07, *P*<0.05) increased the risks of developing DPN (all *P*>0.05) (Table 3).

### Comparisons of CACNA 1A, CACNA 1C, and CACNA 1H genotypes with body mass index, 2 h PG, HbA1c, and TG

In the following study, in order to compare CACNA 1A, CACNA 1C, and CACNA 1H in each group, we conducted this procedure. Individuals carrying the genotype GA + GG had elevated levels of HbA1c and TG than those carrying genotype AA in CACNA 1A rs2248069 in both the DPN and non-DPN groups (both *P*<0.05). Elevated levels of HbA1c and TG were also observed in individuals with the genotype CT + TT in comparison with those carrying the genotype CC in both CACNA 1A rsl6030 and CACNA 1C rs216008 genes (all *P*<0.05). In addition, those carrying the GC + CC genotype also had increased levels of HbA1c and TG levels than those carrying genotype GG in CACNA 1C rs2239050 and CACNA 1H rs7191246 (all *P*<0.05). The body mass index (BMI) and 2 h PG were found without a notable difference between the genotypes in the two groups (all *P*>0.05). Similarly, there was no significant difference observed in the BMI, 2 h PG, HbA1c, and TG of the CACNA 1A, CACNA 1C, and CACNA 1H genotypes in the control group (all *P*>0.05) (Tables 4–6).

**Table 4:** Correlations of CACNA 1A, CACNA 1C, and CACNA 1H genotypes with BMI, 2 h PG, HbA1c, and TG in the DPN group

Genotype	BMI (kg/m^2^)	2 h PG (mmol/l)	HbA1c (%)	TG (mmol/l)
**CACNA 1A**				
rs2248069				
AA	25.59 ± 3.24	14.96 ± 4.29	10.71 ± 3.26	4.69 ± 1.53
GA + GG	25.35 ± 3.33	14.49 ± 4.51	7.83 ± 2.49	2.99 ± 1.31
*P*	0.960	0.603	<0.001	<0.001
rsl6030				
CC	25.57 ± 3.08	14.56 ± 3.93	8.93 ± 3.31	4.01 ± 1.49
CT + TT	25.09 ± 3.55	14.64 ± 5.19	7.92 ± 2.54	2.56 ± 1.24
*P*	0.390	0.913	0.037	<0.001
**CACNA 1C**				
rs216008				
CC	25.53 ± 3.31	14.96 ± 4.10	9.14 ± 3.23	4.01 ± 1.55
CT + TT	25.26 ± 3.30	14.34 ± 4.61	7.89 ± 2.49	2.99 ± 1.41
*P*	0.633	0.459	0.004	<0.001
rs2239050				
GG	25.47 ± 3.32	14.62 ± 4.04	9.15 ± 3.29	4.02 ± 1.47
GC + CC	25.27 ± 3.42	14.47 ± 5.0.	7.92 ± 2.46	2.81 ± 1.39
*P*	0.715	0.945	0.010	<0.001
**CACNA 1H**				
rs3794619				
TT	25.33 ± 3.12	14.65 ± 4.04	9.15 ± 3.29	4.04 ± 1.48
TC + CC	25.39 ± 3.43	14.54 ± 4.96	7.92 ± 2.46	2.83 ± 1.37
*P*	0.923	0.885	0.012	<0.001
rs7191246				
GG	25.48 ± 3.15	14.63 ± 3.89	8.93 ± 3.10	3.98 ± 1.48
GC + CC	25.20 ± 3.52	14.54 ± 5.40	7.85 ± 2.56	2.53 ± 1.24
*P*	0.615	0.908	0.029	<0.001

**Table 5:** Correlations of CACNA 1A, CACNA 1C, and CACNA 1H genotype and allele frequencies with BMI, 2 h PG, HbA1c, and TG in the non-DPN group

Genotype	BMI (kg/m^2^)	2 h PG (mmol/l)	HbA1c (%)	TG (mmol/l)
**CACNA 1A**				
rs2248069				
AA	25.89 ± 3.16	14.68 ± 4.66	11.55 ± 2.85	4.02 ± 1.09
GA + GG	25.01 ± 3.78	14.99 ± 4.30	9.17 ± 2.69	3.62 ± 1.43
*P*	0.192	0.967	<0.001	0.046
rsl6030				
CC	25.60 ± 3.43	14.63 ± 4.63	10.33 ± 3.12	4.20 ± 1.04
CT + TT	24.95 ± 3.61	14.61 ± 3.90	9.08 ± 2.47	2.90 ± 1.46
*P*	0.338	0.929	0.030	<0.001
**CACNA 1C**				
rs216008				
CC	25.38 ± 3.35	14.50 ± 4.62	10.70 ± 3.10	4.27 ± 1.04
CT + TT	25.34 ± 3.70	14.87 ± 4.15	9.00 ± 2.55	3.18 ± 1.41
*P*	0.997	0.684	0.001	<0.001
rs2239050				
GG	25.16 ± 3.41	14.69 ± 4.54	10.45 ± 3.10	4.24 ± 1.04
GC + CC	25.69 ± 3.67	14.61 ± 4.08	9.10 ± 2.59	2.94 ± 1.40
*P*	0.390	0.919	0.008	<0.001
**CACNA 1H**				
rs3794619				
TT	25.35 ± 3.56	14.68 ± 4.55	10.36 ± 3.04	4.21 ± 1.03
TC + CC	25.43 ± 3.47	14.60 ± 3.88	8.95 ± 2.58	2.67 ± 1.37
*P*	0.897	0.920	0.009	<0.001
rs7191246				
GG	25.39 ± 3.46	14.66 ± 4.54	10.19 ± 2.93	4.06 ± 1.18
GC + CC	25.31 ± 3.77	14.65 ± 3.65	8.95 ± 2.97	2.54 ± 1.27
*P*	0.901	0.991	0.039	<0.001

**Table 6:** Correlations of CACNA 1A, CACNA 1C, and CACNA 1H genotype and allele frequencies with BMI, 2 h PG, HbA1c, and TG in the control group

Genotype	BMI (kg/m^2^)	2 h PG (mmol/l)	HbA1c (%)	TG (mmol/l)
**CACNA 1A**				
rs2248069				
AA	26.51 ± 5.28	15.97 ± 2.88	4.79 ± 1.10	2.06 ± 0.57
GA + GG	26.24 ± 5.01	15.62 ± 3.34	4.67 ± 1.21	2.06 ± 0.60
*P*	0.727	0.457	0.492	0.980
rsl6030				
CC	26.48 ± 5.22	15.79 ± 2.96	4.81 ± 1.15	2.04 ± 0.58
CT + TT	26.98 ± 5.08	15.58 ± 3.30	4.48 ± 1.21	2.03 ± 0.56
*P*	0.619	0.946	0.150	0.498
**CACNA 1C**				
rs216008				
CC	26.34 ± 5.35	15.99 ± 2.88	4.82 ± 1.16	2.06 ± 0.57
CT + TT	26.17 ± 5.10	15.37 ± 3.21	4.47 ± 1.19	2.02 ± 0.62
*P*	0.969	0.258	0.195	0.870
rs2239050				
GG	26.44 ± 5.28	15.98 ± 2.90	4.83 ± 1.16	2.07 ± 0.56
GC + CC	26.20 ± 4.89	15.42 ± 3.44	4.53 ± 1.15	2.04 ± 0.59
*P*	0.759	0.263	0.095	0.810
**CACNA 1H**				
rs3794619				
TT	26.20 ± 5.27	15.99 ± 2.89	4.82 ± 1.16	2.05 ± 0.55
TC + CC	26.40 ± 5.19	15.33 ± 3.45	4.45 ± 1.20	2.06 ± 0.63
*P*	0.559	0.199	0.127	0.811
rs7191246				
GG	26.42 ± 5.14	15.81 ± 2.91	4.75 ± 1.21	2.01 ± 0.57
GC + CC	26.11 ± 5.09	15.64 ± 3.82	4.63 ± 0.99	2.23 ± 0.57
*P*	0.747	0.777	0.573	0.052

### Logistic regression analysis of the risk factors of DPN

Based on the logistics regression analysis, DPN were classified as the independent variable, and genotypes in rs2248069, rsl6030, rs216008, rs2239050, rs3794619, and rs7191246 as the dependent variables. Other dependent variables identified include the course of the disease and HbA1c expression, which were of statistical significance in univariate analysis. Based on these results, the course of disease, rs2248069, rsl6030, rs2239050, and rs7191246 polymorphisms were all determined to increase susceptibility to DPN in diabetic patients over a longer period time. This shows that the duration DM was also a risk factor in the development of DPN (all *P*<0.05) (Table 7).

**Table 7:** Multiple logistic regression analysis in the control and DPN groups

Genotype	B	S.E.M.	OR	OR 95% CI	*P*
Course of disease	0.276	0.038	1.317	1.222–1.420	<0.001
HbA1c	–0.082	0.055	0.921	0.826–1.027	0.137
rs2248069	0.883	0.444	2.418	1.012–5.779	0.047
rsl6030	–2.819	0.823	0.06	0.012–0.300	0.001
rs216008	–0.208	0.507	0.812	0.301–2.193	0.681
rs2239050	1.209	0.571	3.349	1.049–10.256	0.034
rs3794619	–0.213	0.626	0.808	0.237–2.756	0.733
rs7191246	2.686	0.742	14.67	3.429–62.768	<0.001

Abbreviation: B, β coefficient.

### Interaction between CACNA 1A, CACNA 1C, and CACNA 1H gene polymorphisms and DPN

Finally, in order to prove the interaction of the CACNA 1A, CACNA 1C, and CACNA 1H gene polymorphisms, the six SNPs of CACNA 1A, CACNA 1C, and CACNA 1H genes were incorporated into the GMDR model as variables. The results obtained indicated that the one-, two-, three-, four-, five- and six-dimensional model combinations were of a statistically significant value (all *P*<0.05). The optimal models were three-, four- and five-dimensional models with the highest prediction accuracy of 61.05% (*P*=0.001) and the best cross-validation consistency was ten out of ten, which indicates the presence of interactions of polymorphisms amongst the CACNA 1A, CACNA 1C, and CACNA 1H genes on DPN (Table 8).

**Table 8:** Interaction effects of SNPs in CACNA 1A, CACNA 1C, and CACNA 1H on DPN in GMDR model

Model dimension	Factor combination	Cross validation consistency	Prediction accuracy (%)	*P*
One	rsl6030	6/10	57.56	0.002
Two	rsl6030/rs2248069	7/10	57.56	0.001
Three	rs216008/rsl6030/rs2248069	10/10	61.05	0.001
Four	rs216008/rs2239050/rsl6030/rs2248069	10/10	61.05	0.001
Five	rs3794619/rs216008/rs2239050/rsl6030/rs2248069	10/10	61.05	0.001
Six	rs3794619/rs7191246/rs216008/rs2239050/rsl6030/rs2248069	10/10	59.88	0.001

## Discussion

DPN is the most common complication that can arise from diabetes and the leading cause of morbidity and mortality in diabetic patients in developed countries [4]. Type 2 diabetes is characterized by an impaired glucose tolerance, which can be prevented with the use of pioglitazone [22]. Nowadays, the treatment of DPN involves the blockade of the renin–angiotensinogen system (RAS) for a high intensity treatment and blood pressure control [23]. In the present study, the relationship between SNPs in the calcium channel genes CACNA 1A, CACNA 1C, and CACNA 1H genes with DPN were investigated amongst the Chinese population. Calcium channels are classified into high voltage activated (HVA) channels which include L-, N-, P-, Q-, and R-types and low voltage activated (LVA) channels which are also known as T-type channels based on their pharmacological profiles and distinct functions [13]. Of the channels, P- and Q-type calcium channels are mainly distributed in the cerebellar Purkinje cells and presynaptic membranes, their pore forming subunit 1A which is encoded by the CACNA 1A gene. The 1C subunit in L-type channels that are located in the postsynaptic dendrites are encoded by the CACNA 1C gene, while the T-type channels are mainly expressed in the cell bodies and dendrites and are encoded by the CACNA 1H gene [24].

The data analysis initially revealed that the prevalence of DPN was closely linked to a variety of indexes including the course of disease, FI, PI, glycemic control, HbA1c, FPG, 2 h PG, and TG. In addition, the course of disease index was also verified to be a risk factor of DPN based on the logistic regression analysis. A study conducted by Lazo et al. [25] demonstrated that the progression of DPN was to some extent determined by the course of DM and HbA1c, which was consistent with the results from the current study. Wile and Toth [26] and Wiggin et al. [27] have also pointed out that both insulin deficiency and elevated fasting TG were important factors contributing to the severity of DPN. This study also showed that there was a notable difference in the levels of HBA1C and TG between AA and GA + GG. HbA1c and TG are ascertained to be closely related with diabetes and increased levels of HbA1c and TG signal can lead to increased possibilities of the development of macrovascular and microvascular lesions in diabetic patients [28,29]. These findings were in accordance with the present results. However, elevated HbA1c and TG levels can also occur as a result of a difference between AA and GA + GG as well as changes of HbA1c and TG levels caused by allele mutations. A certain deviation might have occurred due to the small sample size.

The present study also found that the CACNA 1A, CACNA 1C, and CACNA 1H gene polymorphisms were implicated in the progression of DPN. According to a previous study, there exists a correlation between SNPs and DPN amongst the Chinese population [30]. The association between SNPs and pain pathways have been validated by growing evidence, and the analysis of Meng et al. [31] revealed that SNPs, accompanied by GFRA2 in the Chr8p21.3 might be contributing factors in pain that is associated with diabetic neuropathy. This research gave emphasis to the importance of CACNA in the development of neuropathy. Diabetic microangiopathy includes retinopathy, neuropathy, and nephropathy [32], out of which, retinopathy and nephropathy have been validated to have close association with CACNA in previous studies [33,34]. However, based on a study by Lv et al. [20] rs2248069 and rsl6030 in the CACNA 1A gene and rs3794619 and rs7191246 in the CACNA 1H gene were found without any associations with drug-resistant epilepsy in the Chinese Han population. This study was amongst the few investigations on the same SNPs of the calcium channel genes that were investigated in the present study. Thus, the difference in the correlation of the investigated SNPs between DPN and drug-resistant epilepsy in the Chinese Han population was an interesting point. Unlike DPN, epilepsy is a severe chronic disorder in the brain that is associated with several congenital, genetic, and developmental disorders, which mainly occurs during the period of childhood to early adulthood [35,36]. A previous study has illustrated that the various neurological phenotypes coming from P/Q-type calcium channel dysfunction can affect the calcium ion flow and thus further influence the pathogenetic mechanisms of neurological diseases [37]. CACNA 1C and CACNA 1H are both involved in chronic pain and pain signaling and participate in neurone excitability, neuropathy inflammation, and neurotransmitter release [38]. Moreover, according to Nagi et al. [39], CACNA 1H calcium channels attribute to the abolition of pain in rats with DPN. CACNA 1C rs216008 was also closely linked to cigarette smoking in facilitating the formation of carotid plaque [40] and genetic variation in CACNA 1C rs2239050 was associated with brainstem volume [41].

Complicated traits are regulated by diversified genetic factors working unanimously together and it is widely agreed that these interactions, conduce to different phenotypes in the biological process [42]. Being non-parametric and model-free, multifactor dimensionality reduction (MDR) method is a combinatorial approach in the analysis of gene–gene interaction through the identification of the multilocus models and their associations in case–control studies [43]. In this study, GMDR, an extension of MDR was employed to detect the effects of the SNPs on DPN. The results indicated that six SNPs in the CACNA 1A, CACNA 1C, and CACNA 1H genes, respectively had interaction effects on DPN. The optimal models were three-, four- and five-dimensional models with a prediction accuracy of 61.05% (all *P*=0.001). The best consistency of cross-validation was 10/10.

## Conclusion

DPN is a very complex disease that involves an array of mechanisms. PCR-RFLP was employed in order to analyze primer sequences, Hardy–Weinberg equilibrium to estimate deviations, logistic regression analysis to detect risk factors of DPN and GMDR model for gene–gene interactions. Despite our current efforts for this investigation, the present study had its limitations. First, there is still very little known about the pathogenesis of DPN that is known to be complicated. Although diabetic retinopathy, oxidative stress biomarkers, and vascular risk factors were all identified to be the risk factors for DPN [44], further studies taking more possible factors into consideration are required. Second, the sample size was limited, which may bias the correlation of polymorphisms in the CACNA 1A, CACNA 1C, and CACNA 1H genes with the occurrence of DPN, and rare SNPs in calcium channel genes might have been overlooked. Finally, the racial background of each patient should be taken into consideration since it might lead to different SNP distributions, thereby producing an inconsistency in the results. Thus, a more detailed study with a larger sample size and advanced genetic technology is required to further elucidate the pathophysiology of DPN and its contributing factors. Furthermore, gene–gene and gene–environment interactions also need to be taken under consideration when conducting future studies.

## Abbreviations

BMI: body mass index
CI: confidence interval
DM: diabetes mellitus
DPN: diabetic peripheral neuropathy
FI: fasting insulin
FPG: fasting plasma glucose
GMDR: generalized multifactor dimensionality reduction
HbA1c: hemoglobin A1c
MDR: multifactor dimensionality reduction
OR: odds ratio
PCR-RFLP: PCR-restriction fragment length polymorphism
PG: plasma glucose
PI: postprandial insulin
SNP: single nucleotide polymorphism
TG: triglyceride
2 h PG: 2-h plasma glucose
